# Evaluation of pretreatment ADC values as predictors of treatment response to neoadjuvant chemotherapy in patients with breast cancer - a multicenter study

**DOI:** 10.1186/s40644-022-00501-2

**Published:** 2022-12-09

**Authors:** Alexey Surov, Maciej Pech, Hans-Jonas Meyer, Almir G. V. Bitencourt, Hiroshi Fujimoto, Gabrielle C. Baxter, Gorane Santamaría, Fiona J. Gilbert, Andreas Wienke

**Affiliations:** 1 Department of Radiology and Nuclear Medicine, Otto von Guericke University, Magdeburg, Germany; 2 Department of Diagnostic and Interventional Radiology, University of Leipzig, Leipzig, Germany; 3 Department of Imaging – A.C.Camargo Cancer Center, São Paulo, Brazil; 4 Department of General Surgery, Chiba University Graduate, School of Medicine, 1-8-1 Inohana, Chuo-ku, Japan; 5 Department of Radiology, School of Clinical Medicine, University of Cambridge, Cambridge, UK; 6 Departments of Radiology, Hospital Clínic de Barcelona, University of Barcelona Medical School, Barcelona, Spain; 7 Institute of Medical Epidemiology, Biostatistics, and Informatics, Martin-Luther-University Halle-Wittenberg, Halle, Germany

**Keywords:** Breast cancer, DWI, ADC, Neoadjuvant treatment

## Abstract

**Background:**

Magnetic resonance imaging (MRI) can be used to diagnose breast cancer. Diffusion weighted imaging (DWI) and the apparent diffusion coefficient (ADC) can reflect tumor microstructure in a non-invasive manner. The correct prediction of response of neoadjuvant chemotherapy (NAC) is crucial for clinical routine.

Our aim was to compare ADC values between patients with pathological complete response (pCR) and non-responders based upon a multi-center design to improve the correct patient selection, which patient would more benefit from NAC and which patient would not.

**Methods:**

For this study, data from 4 centers (from Japan, Brazil, Spain and United Kingdom) were retrospectively acquired. The time period was overall 2003–2019. The patient sample comprises 250 patients (all female; median age, 50.5). In every case, pretreatment breast MRI with DWI was performed. pCR was assessed by experienced pathologists in every center using the surgical specimen in the clinical routine work up. pCR was defined as no residual invasive disease in either breast or axillary lymph nodes after NAC. ADC values between the group with pCR and those with no pCR were compared using the Mann–Whitney U test (two-group comparisons). Univariable and multivariabe logistic regression analysis was performed to predict pCR status.

**Results:**

Overall, 83 patients (33.2%) achieved pCR. The ADC values of the patient group with pCR were lower compared with patients without pCR (0.98 ± 0.23 × 10^− 3^ mm^2^/s versus 1.07 ± 0.24 × 10^− 3^ mm^2^/s, *p* = 0.02). The ADC value achieved an odds ratio of 4.65 (95% CI 1.40–15.49) in univariable analysis and of 3.0 (95% CI 0.85–10.63) in multivariable analysis (overall sample) to be associated with pCR status. The odds ratios differed in the subgroup analyses in accordance with the molecular subtype.

**Conclusions:**

The pretreatment ADC-value is associated with pathological complete response after NAC in breast cancer patients. This could aid in clinical routine to reduce treatment toxicity for patients, who would not benefit from NAC. However, this must be tested in further studies, as the overlap of the ADC values in both groups is too high for clinical prediction.

## Background

Breast cancer is a major global health problem and major cause of mortality in women [1]. The average annual female breast cancer incidence rate was 127.3 cases per 100,000 females [2]. Approximately 2.26 million new cases of invasive breast cancer and about 685,000 deaths are expected among women each year worldwide [1].

Imaging plays a major role in diagnosis and treatment planning of breast cancer [3, 4]. The diagnostic imaging gold standard is magnetic resonance imaging (MRI), which combines a high sensitivity and specificity to diagnose breast cancer and has superior accuracy compared to mammography and ultrasonography. It is especially important for surgical and radiation treatment planning.

The MRI protocol is not clearly standardized and there are sequence differences throughout the imaging centers. In daily clinical practice, MRI utilizes T2-weighted with or without fat saturation and dynamic-contrast enhanced T1-weighted sequences [3]. The addition of diffusion-weighted imaging (DWI) can be used as a functional imaging. DWI can be quantified by apparent diffusion coefficient (ADC) [5, 6]. There are still concerns with DWI in clinical routine with not yet standardized imaging technique with different b-values and the proneness for artefacts.

The diagnostic and prognostic benefits of ADC values have been shown in various tumor entities throughout oncologic imaging, including breast cancer [7, 8]. Importantly, ADC inversely correlates with cellularity and/or proliferation activity in different tumors [5]. As such, ADC can discriminate benign from malignant breast lesions [9]. With this approach, it was shown that the use of DWI in the diagnosis of breast cancer can reduce the number of biopsies by approximately 20% due to the correct categorization of benign tumors with a high ADC-value above 1.2 × 10^− 3^ mm^2^/s [10]. Moreover, a low value of less than 1.0 × 10^− 3^ mm^2^/s was reported to be highly suspicious for malignancy [9].

Furthermore, some authors suggested that ADC can be used for prediction of treatment response to neoadjuvant therapy [11, 12]. So far, Fangberget et al. proposed a cut off value of 1.42 × 10^− 3^ mm^2^/s [11]. Using this cut off, pCR can be predicted with sensitivity and specificity of 88% and 80%, respectively [11].

However, there were also negative reports regarding the possible use to predict treatment response to neoadjuvant treatment. For instance, a large meta analysis could not identify relevant differences between tumors with pathological complete response (pCR) and those without utilizing the pretreatment ADC value [13]. Yet, there are also reports that an increase of the ADC value during neoadjuvant therapy might be a valuable biomarker to correctly predict the patients with pCR [14]. Furthermore, the utilization of only the pretreatment ADC value would benefit patients to reduce possible treatment toxicity, which might not be needed in non-responders. Yet, the current most valuable aspect of ADC values is the increase under treatment in follow-up investigations to clearly depict a treatment response.

However, there is still lack of standardisation of ADC values in regard of sequence parameters, scanner technology and ADC measurement technique, which need to be addressed before ADC values are an additional biomarker in clinical routine [7]. This is also one reason why DWI and ADC values are not included into the BIRADS catalogue to date. Previous studies used ADC values to predict pCR status predominantly in single center designs with small patient samples, which reduces possible external translation due to the abovementioned limitations of ADC values. That is why there is need to test the possible clinical benefit in a multi-centric setting.

Therefore, the present study analyzed the pretreatment ADC values to predict breast cancer patients with pCR after neoadjuvant therapy based upon a large patient sample and whether there are differences regarding the molecular subtype of the breast cancer patients.

## Methods

### Patients and tumors

This study was approved by the institutional review board of the central study center (Ethic Commission of the Medical Faculty, Otto-von-Guericke University Magdeburg, Nr. 36/20). For each center there was an ethical approval regarding the investigation of breast MRI in patient undergoing NAC.

Overall, 250 patients (all females; median age, 50.5; range, 27–84 years, convenience sample) were included in the study. The data was comprised from the following centers: Center 1: A.C. Camargo Cancer Center, São Paulo, SP, Brazil; Center 2: Department of Radiology, Chiba University Graduate School of Medicine, Chiba, Japan; Center 3: Department of Radiology, School of Clinical Medicine, University of Cambridge, Box 218, Cambridge Biomedical Campus, Hills Road, Cambridge CB2 0QQ, UK; Center 4: Departments of Radiology, Hospital Clínic de Barcelona and University of Barcelona Medical School, Barcelona, Spain.

The patients were retrospectively analyzed in the centers including the image analysis. Central statistical analysis was performed. All patients were newly diagnosed breast cancer patients without any previous treatment.

The receptor status of the acquired breast carcinomas was classified according to the St. Gallen consensus meeting [15]. Luminal A carcinomas (hormone receptor positive carcinomas with a Ki-67 expression below 14%) were diagnosed in 41 patients (16.4%), Luminal B carcinomas (hormone receptor positive tumors with a Ki-67 expression over 14%) in 75 patients (30.0%), HER2-enriched carcinomas in 69 cases (27.6%), and triple negative carcinomas in 65 patients (26.0%).

Pathological differentiation of the carcinomas was as follows: well-differentiated (grade 1) was diagnosed in 13 cases (5.2%), moderately differentiated (grade 2) in 118 cases (47.2%) and poorly differentiated (grade 3) tumors in 107 cases (42.8%). In 12 cases no information regarding the differentiation was provided (4.8%).

Furthermore, the included tumors were staged as T1 in 28 cases (10.9%), T2 in 162 cases (64.8%), T3 in 53 cases (21.2%), and T4 in 7 cases (2.8%). T-stage was obtained before the treatment.

There were no tumors with distant metastases (M1 stage), as was confirmed by clinical routine within the centers.

In all cases, MRI with DWI was performed on 1.5 or 3.0 T clinical scanners with dedicated breast radiofrequency coils. Table 1 provides information regarding the utilized sequence parameters. MRI was performed in all centers before any form of treatment. NAC was chosen as a therapeutic avenue for selected high-risk breast cancers, tumours ≥2 cm and for locally advanced (including initially ineligible for resection) disease.

**Table 1:** Imaging parameters in the centers

Centers	MR scanners	DWI sequence technique
1	2 different 1.5 T scanners: GE Medical (Milwaukee, WI, USA) and Achieva, Philips Healthcare, (Best, Netherlands)	FOV 160 × 192 mm slice thickness 5 mm TR 4200 ms TE 85 ms b-values 0, 250, 500, 750 and 1000 s/mm^2^
2	1.5 T Intera Achieva (Philips Medical Systems, Best, The Netherlands	FOV 360 × 216 mm slice thickness 5 mm TR 3783 ms TE 64 ms b-values: 0 and 800 s/mm^2^.
3	3.0-T system (MR750, GE Healthcare)	FOV 350 × 350 mm Slice thickness 4 mm TR 5 ms TE 77.9 ms b-values: 0, 30, 60, 90, 120, 300, 600 and 900 s/mm^2^
4	2 different 1.5 T system (Signa; GE Medical Systems, Milwaukee, Wis, Aera; Siemens, Erlangen, Germany)	FOV 320 × 320, and 360 × 270 mm Slice thickness 4 mm TR 8000 and 6500 ms TE 65 and 66 ms b-values: 0 and 700 s/mm^2^ and 50 and 700 s/mm^2^

### ADC values

The ADC values were measured on the ADC map with a region of interest (ROI) on the representative slide within the tumor boundaries defined by T1-weighted contrast enhanced images. The ROI was standardized placed within the whole tumor within the tumor boundaries. The measurements were performed in each center by an experienced radiologist.

### Neoadjuvant chemotherapy regimen

Neoadjuvant chemotherapy regimen differed between the included centers. In center 1, the regimen consisted of four cycles of anthracycline and cyclophosphamide at intervals of 3–4 weeks followed by four cycles of weekly paclitaxel (AC-T) (*n* = 37). Trastuzumab was added to this regimen (AC-T) in the 16 cases with HER-2 overexpression. In center 2, neoadjuvant chemotherapeutic regimens consisted of adriamycin 60 mg/m^2^ and cyclophosphamide 600 mg/m^2^ once every 3 weeks, followed by 12 weekly doses of paclitaxel 80 mg/m^2^ (*n* = 18) and 4 cycles of 5-fluorouracil 500 mg/m^2^, epirubicin 100 mg/m^2^, and cyclophosphamide 500 mg/m^2^ (FEC100) once every 3 weeks, followed by 4 cycles of paclitaxel 175 mg/m^2^ once every 3 weeks (*n* = 38). In center 3, The regimen consisted of docetaxel 100 mg/m^2^ once every 21 days for three cycles, followed by fluorouracil 500 mg/m^2^, epirubicin 100 mg/m^2^, with cyclophosphamide 500 mg/m^2^ (FEC) once every 21 days for three cycles if the tumour was negative for human epidermal growth factor 2 (HER2−) on biopsy. Lastly, in center 4, HER2-negative tumors, either ER positive or ER negative, were treated with six cycles of anthracycline and taxane in combination over 5–6 months. In HER2-positive tumors, trastuzumab was added to the chemotherapy. The time interval between NAC and surgical therapy was 3 months.

### pCR

pCR was assessed by experienced pathologists in every center using the surgical specimen in the clinical routine work up. PCR was defined as no residual invasive disease in either breast or axillary lymph nodes after NAC. PCR was defined in accordance to the Residual Cancer Burden (RCB) protocol in three centers [16] and in accordance to the Japanese Breast Cancer Society criteria [17] in one center.

### Statistical analysis

For statistical analysis the SPSS statistical software package (SPSS 25, SPSS Inc., Chicago IL, USA) was used. Continuous variables were described using mean, median and standard deviation. Categorical variables were given as absolute and relative frequencies. ADC values between different groups were compared using the Mann–Whitney U test (two-group comparisons). Correlation analysis was performed with Pearson’s correlation coefficient. Univariable and multivariable logistic regression analysis was performed.

## Results

### Pretreatment ADC values

The pretreatment mean ADC value in the whole patient sample was 1.037 ± 0.24 × 10^− 3^ mm^2^/s, range 0.36–2.0 × 10^− 3^ mm^2^/s. Table 2 provides ADC values in accordance to the tumor molecular subtypes. Luminal B carcinomas had lower ADC values compared with Luminal A (*p* = 0.025), HER2-enriched carcinomas (*p* = 0.004) and with triple negative carcinomas (*p* = 0.004). No relevant differences were identified between Luminal A, HER2-enriched and triple negative carcinomas.

**Table 2:** Pretreatment ADC values according to the molecular subtypes

	Overall patient sample	Luminal A	Luminal B	HER2-enriched	Triple negative
**ADC-values (10**^**− 3**^ **mm**^**2**^**/s, M ± SD**	1.04 ± 0.24	1.05 ± 0.18	0.93 ± 0.21	1.07 ± 0.22	1.10 ± 0.29

There was no correlation between ADC and T-Stage (*r* = 0.04, *p* = 0.50, Pearson’s correlation analysis). A weak inverse correlation was identified between ADC and histopathological tumor grade (*r* = − 0.14, *p* = 0.03).

### pCR status

In the overall study sample, 83 patients (33.2%) achieved pCR. In the Luminal A subgroup, only 2 tumors (5%) achieved pCR and in 38 tumors (95%) no pCR was found. In the Luminal B subgroup, in 11 cases (14.7%) pCR was diagnosed and in 63 lesions (85.3%) residual tumor was identified. In the HER2-enriched subgroup, 34 tumors (49.3%) achieved pCR and in 35 cases (51.7%) residual tumors were detected. Finally, in the triple negative subgroup, in 36 lesions (55.4%) pCR and in 29 lesions (44.6%) residual tumors were diagnosed.

### Associations between ADC and pCR

**Fig. 1:**
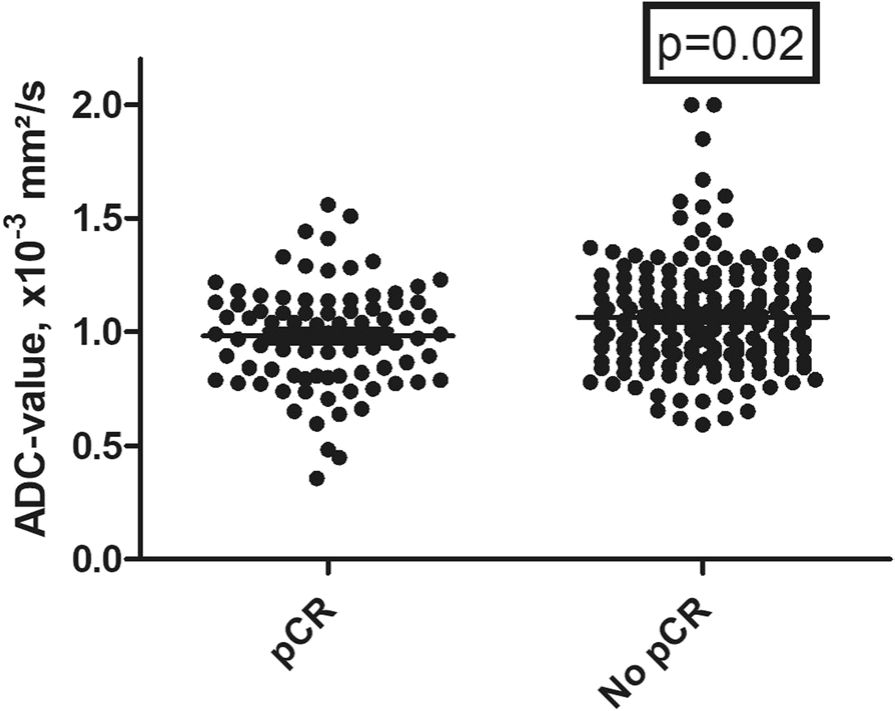
Scatter plot of the ADC-values in accordance to pCR and non pCR. The tumors with pCR showed lower ADC values compared to the lesions with non pCR

The ADC values of the patient group with pCR were lower compared with patients without pCR (0.98 ± 0.23 × 10^− 3^ mm^2^/s versus 1.07 ± 0.24 × 10^− 3^ mm^2^/s, *p* = 0.02). Figure 1 displays the corresponding scatter plot.

In regression analysis the ADC value achieved an odds ratio of 4.65 (95% CI 1.40–15.49) in univariable analysis and of 3.0 (95% CI 0.85–10.63) in multivariable analysis in the overall patient sample (Table 3). The odds ratios differed in the subgroup analyses in accordance with the molecular subtype (Table 3). The highest odds ratio was achieved for the HER2 enriched subtype.

**Table 3:** Uni- and multivariable analysis to predict pCR in the tumors based on the pretreatment ADC values

**Univariable analysis**			
**Tumors**	**Odds ratio**	**95% CI**	***p*-value**
Overall sample	4.65	1.40–15.49	0.012
HER2-enriched tumors	29.12	1.95–434.22	0.014
Triple negative tumors	15.78	1.71–145.53	0.015
Luminal B tumors	35.81	1.19–1075.04	0.039
**Multivariable analysis**^a^			
**Tumors**	**Odds ratio**	**95% CI**	***p*-value**
Overall sample	3.00	0.85–10.63	0.088
HER2-enriched tumors	76.98	2.75–2154.73	0.011
Triple negative tumors	19.20	0.96–382.16	0.053
Luminal B tumors	54.86	1.26–2390.30	0.038

^a^adjusted for T stage and histological grading

## Discussion

The present study investigated the ability of pretreatment ADC to predict pCR in breast cancer patients undergoing neoadjuvant chemotherapy. The prediction of treatment outcome could be very important for clinical care. In fact, accurate prediction of treatment response using imaging could help to individualize treatment and to avoid ineffective chemotherapy in patients.

A key finding of the present study was the dependence of the molecular subtype for the association between pCR and pretreatment ADC values. In fact, pCR is the best outcome for neoadjuvant chemotherapy in breast cancer patients [18, 19]. As reported previously, it is an important prognostic factor for both disease-free survival and overall survival in patients with breast cancer [18, 19]. So far, patients with pCR of breast cancer have an improved 5-year disease-free survival rate of 87% and a 5-year overall survival rate of 89% in comparison to patients without pCR [20].

Previously, a meta analysis including 13 studies with 575 patients identified that the pooled sensitivity and specificity of MRI in prediction of pCR was 0.88 (95% CI, 0.78; 0.94) and 0.69 (95% CI, 0.51; 0.83), respectively [21]. The included studies used morphological MRI data including T2 weighted and dynamic contrast enhanced (DCE-MRI) images but not DWI. In another meta analysis investigating only studies with DCE-MRI, a pooled sensitivity of 0.80 (95% CI, 0.70, 0.88) and a specificity of 0.84 (95% CI; 0.79, 0.88) was identified [22]. While the sensitivity can be considered as sufficient for clinical routine, there is still lack of specificity. Presumably, the addition of another diagnostic MRI sequence might improve diagnostic results.

The present study provides new data regarding the subtypes as well as multivariable analyses, which were not as clearly stated before. It was shown that ADC values have the highest statistical association with pCR prediction in the HER2 enriched subtype, which might be caused by the different treatment regimes for this subtype including the addition of trastuzumab. However, the exact underlying reasons for this behaviour remain elusive.

There are several reasons why ADC values might be able to predict pCR. Ideally, neoadjuvant chemotherapy reduces tumor cell count completely. Biologically aggressive tumors particularly benefit from neoadjuvant chemotherapy, as tumors are more vulnerable to chemotherapy when in a proliferation state. ADC correlates inversely with cell count and tumor aggressiveness [6, 9, 23]. The direct inverse associations between ADC values and proliferation potential, quantified by Ki-67 index was shown in several analyses, including for breast cancer patients [24–27].

It has already been shown that the ADC is a valuable imaging parameter to discriminate benign from malignant tumors, as the proposed threshold of 1.0 × 10^− 3^ mm^2^/s was identified in a large meta analysis based upon 13,847 lesions [9]. In another recent study, ADC values were also capable of reducing biopsies in BIRADS 4 lesions in up to 32.6% of cases [10].

One key fact is that ADC values increased during/after neoadjuvant chemotherapy as another important discriminating parameter to assess treatment response [14, 27]. Numerous studies confirmed this hypothesis including a large multicenter trial based in North America in a prospective setting [14]. Moreover, increase of ADC values during neoadjuvant chemotherapy was more useful than tumor size or volume change after therapy [28]. As such, most studies utilized the difference between the pretreatment ADC value and the ADC value after treatment to predict pCR.

However, a more important question is whether it is possible to predict the effect of neoadjuvant chemotherapy accurately based on pretreatment values. The reported data using pretreatment ADC are contradictory [13]. While some authors found an association between pretreatment ADC and pCR after neoadjuvant chemotherapy, others did not. Bedair et al. reported that responders had lower pretreatment ADC values (× 10^− 3^ mm^2^/s) in comparison to non-responders, namely 0.92 ± 0.02 and 1.20 ± 0.02, respectively (*p* < 0.001) [29]. Similar results were reported by Liu et al. based upon a large retrospective study with 176 patients [30]. In this study different cut-off values were also proposed in accordance with the molecular subtype. Thus, triple negative cancers had the highest ADC-cut off value with 1.43 × 10^− 3^ mm^2^/s, whereas Luminal B had the lowest with 1.33 × 10^− 3^ mm^2^/s [30]. One reason for the identified results for treatment response prediction could be seen in these reported inherent differences of ADC values according to the subtypes. Yet, there is definite need for further research in this regard.

However, in the study of Bufi et al. there were no relevant differences of pretreatment ADC values between responders and non-responders: 1.13 ± 0.19 vs 1.09 ± 0.19 (× 10^− 3^ mm^2^/s), respectively, concluding that pretreatment ADC values are not a useful imaging parameter to predict treatment response [31]. Of note, in this study, the most patients had the Luminal A subtype with 143 of 225 patients [31].

In short, the results of using pretreatment ADC values to reliably predict treatment response following neoadjuvant chemotherapy are still conflicting. One strength of our present analysis is the multivariable regression analysis to adjust for potential confounders, which was not performed previously. A recent study employed a multivariable regression analysis to predict pCR based upon 50 patients [32]. While clinical stage and T stage had high associations with pCR, for MRI findings, only the ADC value change below 15% after two cycles of chemotherapy was associated with pCR (OR= 9.865, 95%CI 1.024–95.021) [32].

Choi et al. investigated a novel ADC-parameter, called ADCdiff, which is the difference between the maximum and minimum ADC values [33]. With this approach, the ADCdiff was superior to ADCmean, ADCmax and ADCmin to predict pCR in this study based on 49 patients [33].

The present analysis based on a multicenter cohort showed that pretreatment ADC values are an independent parameter associated with pCR but the baseline ADC values of responders to NAC and non-responders overlapped in a relevant manner. This was also shown for the subgroups, which suggests that pretreatment ADC cannot be used as a reliable prognostic surrogate marker for pCR.

Our study used subgroup analyses to test for differences with regard to the immunohistochemical subtype. The correct classification of subtype is crucial due to differences in approach to treatment as well as prognostic implications.

One noteworthy study used ^18^F-labeled fluorodeoxyglucose positron emission tomography-computed tomography (PET-CT) to predict the response to trastuzumab or pertuzumab in HER2-positive breast cancer [34]. The primary endpoint was however not met and the area under the curve was only 0.72 (80% CI, 0.64 to 0.80) [34]. One can conclude that even FDG-PET-CT is not able to reliably predict treatment response in the HER-2-enriched subtype.

The present results are based upon large multi-center data, which identified distinctive differences of the associations between pretreatment ADC values and treatment response accordingly to the molecular subtype. Further prospective studies are needed to assess the discriminative power of pretreatment ADC values.

Our analysis has some limitations to address. First, the multi-center cohort was acquired in a retrospective manner with possible inherent bias. Second, the image reading was not performed in a centrally reading session. It was performed in each center by experienced radiologists. There might be some bias obtained by the different readers. However, it was shown that ADC is a reliable imaging biomarker with low interreader heterogeneity and high reproducibility [35, 36]. Third, the centers used different MRI scanners and different DWI sequences, which results in heterogeneity of the ADC values. Beyond that, an important point is that the diffusion time of the included DWI sequences differed between the centers, which could not be accounted for. Standardisation must be achieved to establish ADC values as an imaging biomarker into clinical routine. Fourth, pCR was defined using different classification criteria. This might result in bias but it represents daily clinical care, as these classifications are used in the different centers in clinical routine.

## Conclusion

The pretreatment ADC is associated with pathological complete response after neoadjuvant therapy in breast cancer patients. This could aid in clinical routine to reduce treatment toxicity for patients, who would not benefit from NAC However, this must be tested in further studies, as the overlap of the ADC values between the groups is too high for clinical prediction.

## Data Availability

The data that support the findings of this study are available from professor Surov but restrictions apply to the availability of these data, which were used under license for the current study, and so are not publicly available. Data are however available from the authors upon reasonable request and with permission of professor Surov.

## Abbreviations

pCT: Pathological complete response
ADC: Apparent diffusion coefficient
PET-CT: Positron emission tomography-computed tomography
NAC: Neoadjuvant chemotherapy
